# Advancing Bag-of-Visual-Words Representations for Lesion Classification in Retinal Images

**DOI:** 10.1371/journal.pone.0096814

**Published:** 2014-06-02

**Authors:** Ramon Pires, Herbert F. Jelinek, Jacques Wainer, Eduardo Valle, Anderson Rocha

**Affiliations:** 1 Institute of Computing, University of Campinas (Unicamp), Campinas, São Paulo, Brazil; 2 Department of Biomedical Engineering, Khalifa University, Abu Dhabi, United Arab Emirates, and Australian School of Advanced Medicine, Macquarie University, North Ryde, New South Wales, Australia; 3 School of Electrical and Computing Engineering, University of Campinas (UNICAMP), Campinas, São Paulo, Brazil; Medical University of South Carolina, United States of America

## Abstract

Diabetic Retinopathy (DR) is a complication of diabetes that can lead to blindness if not readily discovered. Automated screening algorithms have the potential to improve identification of patients who need further medical attention. However, the identification of lesions must be accurate to be useful for clinical application. The bag-of-visual-words (BoVW) algorithm employs a maximum-margin classifier in a flexible framework that is able to detect the most common DR-related lesions such as microaneurysms, cotton-wool spots and hard exudates. BoVW allows to bypass the need for pre- and post-processing of the retinographic images, as well as the need of specific *ad hoc* techniques for identification of each type of lesion. An extensive evaluation of the BoVW model, using three large retinograph datasets (DR1, DR2 and Messidor) with different resolution and collected by different healthcare personnel, was performed. The results demonstrate that the BoVW classification approach can identify different lesions within an image without having to utilize different algorithms for each lesion reducing processing time and providing a more flexible diagnostic system. Our BoVW scheme is based on sparse low-level feature detection with a Speeded-Up Robust Features (SURF) local descriptor, and mid-level features based on semi-soft coding with max pooling. The best BoVW representation for retinal image classification was an area under the receiver operating characteristic curve (AUC-ROC) of 97.8% (exudates) and 93.5% (red lesions), applying a cross-dataset validation protocol. To assess the accuracy for detecting cases that require referral within one year, the sparse extraction technique associated with semi-soft coding and max pooling obtained an AUC of 94.2
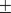
2.0%, outperforming current methods. Those results indicate that, for retinal image classification tasks in clinical practice, BoVW is equal and, in some instances, surpasses results obtained using dense detection (widely believed to be the best choice in many vision problems) for the low-level descriptors.

## Introduction

For progressive diseases, such as complications of *diabetes mellitus*, early diagnosis has a huge impact on prognosis, allowing corrective or palliative measures before irreversible organ damage takes place. In the case of Diabetic Retinopathy (DR), early detection is crucial to prevent vision loss. Therefore, screening patients for early signs of DR pathology is important to prevent the disease or limit its progression. However, in disfavored, rural or isolated communities, the access to healthcare professionals – particularly to ophthalmology specialists – is difficult or not possible, therefore reducing opportunities for early detection and timely treatment of DR.

Computer-aided diagnosis may solve that dilemma by automatically deciding who should be referred to an ophthalmologist for further investigation. However, in order to be useful, the automated system must identify a specific type of lesions that occurs both in isolation and in combination with other types of lesions, and make accurate decisions on the need to refer the patient to a specialist for further assessment.

Most detection algorithms explore specific structural characteristics of a single type of DR lesion. Thus each method is specifically developed for a type of lesions. Those algorithms, therefore, require extensive image pre-processing and many *ad hoc* decisions [1]–[14]. As each algorithm is limited to dealing with a specific lesion, the system must employ a separate algorithm for each DR-related lesion present in the image and combine the results of very distinct algorithms in order to make a decision on referral.

The methodology we propose is based upon the bag-of-visual-words (BoVW) model and employs a different strategy, associating a two-tiered image representation to maximum-margin support-vector machine (SVM) classifiers. Such methodology was widely explored for general-purpose image classification, and consists of the following steps: (i) extraction of low-level local features from the image; (ii) learning of a codebook using a training set of images; (iii) creation of the mid-level (BoVW) representations for the images based on that codebook; (iv) learning of a classification model for one particular lesion using an annotated training set; (v) using the BoVW representation and the learned classification model for deciding on whether or not a specific lesion is present in a retinal image. One advantage of this very flexible framework is that it can employ the same scheme for all lesions, varying only the data used to learn the codebook and the classification model.

The two-tiered image representation rests upon the extraction of low-level local features and their aggregation into mid-level BoVW representation. The mid-level BoVW consists of two operations: the *coding* of the low-level feature vectors using the codebook, and the *pooling* of the codes, which are combined into a single aggregated feature vector [15]. There are several options available for the coding and pooling operations.

The work presented here extends prior work that considered BoVW for detecting DR-related lesions in retinal images [16]–[18] by systematically exploring several alternatives for both the low-level and mid-level feature extraction of two large datasets and performing a full statistical analysis in order to determine the best combination of parameters for classification of DR.

This work focuses on the problem of screening and identification of diabetic retinopathy rather than on evaluating diabetic retinopathy severity. An important contribution of our work is the **decision for** ***referral***. The decision for referral is estimated directly from a normalized vector of the “probability of presence” (confidence scores) of the individual lesions (as assigned by the individual lesion detectors) without the need for an intermediate severity estimation step. The criterion for judging the performance of the automatic referral algorithm is whether it agrees with a set of medical specialists on the need that the patient see an ophthalmologist in the 12 months that follow the fundus image assessment. This work brings important contributions to the empirical evaluation of DR-related lesion detection, in order to make the evaluation more rigorous. First, is the use of a cross-dataset protocol, an important precaution in the design, since in clinical practice, the images that need to be classified have rarely the same image specification (camera, resolution, operator, field of view (FOV)) than the images used for training. Second, is the use of a global statistical analysis to establish the parameters for the BoVW model that achieve the most positive overall effect on improving detection rates.

### Diabetic Retinopathy

Diabetes mellitus is a chronic end-organ disease that affects the circulatory system, including the blood vessels of the retina, where it may trigger diabetic retinopathy (DR). DR is the major cause of blindness for people of working age in Europe and the U.S. Due to the long asymptomatic phase of DR, retinal vascular complications may already be widespread when diagnosis is finally established, compromising the treatment outcomes [19].

As of 2012, diabetes affected 347 million people worldwide [20]. According to the International Diabetes Federation (http://www.idf.org/diabetesatlas/5e/diabetes), prevalence may reach 552 million people by the year 2030. Since the number of ophthalmologists is not increasing at the same rate, there is concern that medical personnel will be unable to cope with the increasing number of DR patients. Therefore, automated screening appears as an important adjunct for diabetes clinics by reducing specialist workload [3], [21], [44]. Automated detection and referral information is particularly important for poor, isolated, or rural communities, where the full-time presence of an ophthalmologist is not possible.

The current state of the art on aided diagnosis of DR, although obtaining a high sensitivity and specificity, tends to be specialized for a specific type of lesion [1]–[14]. For bright lesion detection, sensitivities range from 70.5 to 100.0% and specificities from 84.6 to 99.7% [1], [4], [10]–[14]; for red lesion detection, sensitivities range from 77.5 to 97.0% and specificities from 83.1 to 88.7% [1]–[3]. A summary of results found in the literature is presented in Tables 1 and 2.

**Table 1:** State of the art for the detection of bright lesions.

Work	Sens	Spec	AUC	Dataset	Approach
Sinthanayothin et al. [1]	88.5%	99.7%	–	30	Recursive Region-Growing Segmentation (RRGS) and thresholding
Niemeijer et al. [4]	95.0%	88.0%	95.0%	300	Each pixel is classified in a so-called lesion probability map.
Sánchez et al. [10]	100%	90.0%	–	80	Mixture models and dynamic threshold for segmentation, followed by a postprocessing to distinguish the lesions.
Giancardo et al. [11] ****	–	–	88.0%	169*+1200**+89***	Features based on color, wavelet decomposition and exudate probability. Several classification algorithms.
Fleming et al. [12]	95.0%	84.6%	–	13219	Multi-scale morphological process followed by thresholding
Sopharak et al. [13]	80.0%	99.5%	–	60	Mathematical morphology methods followed by thresholding
Welfer et al. [14]	70.5%	98.8%	–	89***	Mathematical morphology methods and thresholding

*HEI-MED dataset.

**MESSIDOR dataset.

***ROC dataset.

****AUC obtained for training on HEI-MED dataset and test on Messidor dataset.

**Table 2:** State of the art for the detection of red lesions.

Work	Sens	Spec	AUC	Dataset	Approach
Sinthanayothin et al. [1]	77.5%	88.7%	–	23	RGGS in green channel.
Jelinek et al. [2]	97.0%	88.0%	–	758	A microaneurysms (MA) detector notes the number of MAs and dot-hemorrhages detected
Fleming et al. [3]	85.4%	83.1%	90.1%	1441	MA detection with emphasis on the role of local contrast normalization
Giancardo et al. [5] *	–	–	–	100**	Microaneurysms Detection with Radon Cliff Operator
Antal & Hajdu [6]	–	–	90.0%	1200*+100**	Combination of internal component of MA detectors
Lazar & Hajdu [7] *	–	–	–	100**	Statistical measures of attributes on peaks are used in a naïve Bayes classification. Scores are thresholded for a binary output
Zhang et al. [8] *	–	–	–	100**	Multiscale Correlation Filtering (MSCF) and dynamic thresholding for intensity-based detection and localization
Sánchez et al. [9] *	–	–	–	100**	Statistical approach based on mixture model-based clustering and logistic regression

*MESSIDOR dataset.

**ROC dataset.

The development and implementation of single-lesion algorithms is a limitation for accurate referral as, in general, a method developed for one lesion cannot be directly applied to other lesions, preventing the development of a general framework for multi-lesion detection and referral. In order to overcome this, several multi-lesion schemes were proposed. Li et al. [22] implemented a real-time management tool for diabetic eye disease that focuses on the two main DR-related lesions: microaneurysms and hard exudates. However, their framework does not exploit a unique technique for the detection of both lesions simultaneously. Lesions are first detected using several image analysis criteria including texture measurements. This provides a content-based image retrieval framework once the microaneurysms and exudates have been detected in each image. The information is grouped together and a complete description of the retinal image is created as query, which is then compared to a database of past images with known diagnoses.

Another common limitation of using current algorithms for DR detection and classification is the need for complex and *ad hoc* pre- and post-processing of the retinal images, depending on the lesion of interest. The pre- and post- processing address issues like image acquisition and field-of-view variations, or adaptations to take ethnicity of the patients into account [21], [23]. Preprocessing of retinal images may include standardizing the resolution of the image, normalizing color, segmenting and removing blood vessels [24], and detecting and removing the optic disk [3], [25]. For this task, morphological operators [26] are often employed as part of the pre-processing step [12]–[14].

Automated techniques for DR detection are not restricted to the detection of lesion *types* but can also be aimed at identification of the disease *stage/severity*. Nayak et al. [27], for example, used morphological operations and texture analysis to extract the features for an automated classification algorithm with neural networks. The features are related to the area of blood vessels, area of hard exudates and image texture. The neural network then classifies the images as non-proliferative retinopathy, proliferative retinopathy, or normal. A simple scheme for classification of DR progression ranging from healthy to mild, moderate and severe non-proliferative retinopathy was proposed by Jelinek et al. [28] based upon the colorization of the optic disc. Yun et al. [29] also used morphological operations and neural networks for the identification of DR progression. The process begins with contrast improvement, histogram equalization, morphological operations and binarization. After preprocessing the images with morphological operations, the system extracts six features by counting the pixels contained in the perimeter and the area of interest for each RGB channel. Four groups can be identified using this procedure: normal retina, moderate non-proliferative retinopathy, severe non-proliferative retinopathy and proliferative retinopathy. Both methods [27], [29] achieved a sensitivity of 90.0% and a specificity of 100.0% for retinopathy classification.

The decision process for referring or not referring a patient using individual lesion classifiers has also received attention [41]–[44]. Niemeijer et al. [4] combined different detectors for specific lesions into a single automatic decision scheme. More recently, Jelinek et al. [18] also investigated fusion schemes for obtaining decisions from the evidence of specific anomaly detectors.

Research in automated retinal lesion classification is becoming more general, bypassing the need for pre- and post-processing. Rocha et al. [16] proposed a unified framework for detection of both hard exudates and microaneurysms. The authors introduced the use of bags-of-visual-words representations for DR-related lesion detection, creating a framework easily extendible to different types of retinal lesions. However, the bags-of-visual-words model employed in that work was simple and chosen without any theoretical or experimental design analysis but rather from experimental results in other fields of image analysis [30]. This has opened up the opportunity for substantial improvements, which are explored in this paper. Furthermore, alternative combinations for the bags of visual words were evaluated in a more statistically rigorous experimental design, supporting the claims and decisions based on bags of visual words for DR detection and *referral* versus *non-referral* classification.

### BoVW Representations

A two-tiered feature extraction scheme, based upon the creation of an aggregation of encoded local features became a staple of the image classification literature. The technique was popularized by the work of Sivic and Zisserman [30], who made explicit an analogy with the traditional bag-of-words representation used in information retrieval [31]. That formalism from information retrieval is reformulated for local image descriptors as “visual words” by associating the low-level local features to the elements of a codebook, which is aptly named a “visual dictionary”. The number of visual words for a given image is represented as a histogram named bag of visual words (BoVW), and used as a mid-level representation.

Learning the codebook is a challenge for BoVW representations. The traditional way involves unsupervised learning over a set of low-level features from a training set of images. K-means clustering, for example, can be used on a sample of these features and the *k* centroids be employed as codewords. There is also considerable variation throughout the literature on the *size* of the codebook, ranging from a few hundred codewords up to hundreds of thousands.

The metaphor of “visual word” should not be taken too literally. While textual words are intrinsically semantic, visual words are usually appearance-based only. Moreover, the BoVW model was considerably extended since the seminal work of Sivic and Zisserman. New ways of encoding the local descriptors using the codebook were proposed, as well as new ways of aggregating the obtained codes. This stretched the metaphor of “visual word” too much, and a more formal model was proposed by Boureau et al. [15], making the *coding* and *pooling* operations more explicit. Therefore, the BoVW formalism evolved into a meta-model for which myriads of variations are possible, based upon the combinations of low-level descriptors, codebook learning, coding and pooling.

**Figure 1:**
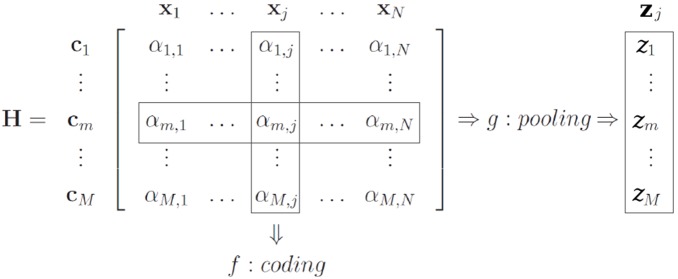
The BoVW model illustrated as a matrix. The figure highlights the relationship between the low-level features **x** *_j_*, the codewords **c** *_m_* of the visual dictionary, the encoded features *α_m_*, the coding function *f* and the pooling function *g*.

The coding and pooling operations can be conveniently understood in matrix form proposed by Precioso and Cord (see Figure 1, adapted from [32], [33]). Their formalism starts with the choice of the codebook (e.g., by sampling or learning on the low-level feature space) as an indexed set of vectors,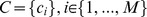, where 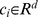. Then, the low-level local features for each image, which are represented by the index set 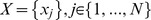, where 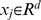 is a local feature and 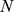 is the number of salient regions, points of interest, or points in a dense sampling grid on the image are extracted. The final BoVW vector representation encodes a relationship between 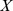 and 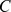 [15], [33].

The coding step transforms the low-level descriptors into a representation based upon the codewords, which is better adapted to the specific task and preserves relevant information, while discarding noise. Coding can be modeled by a function 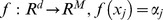 that takes the individual local descriptors 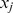 and maps them onto individual codes 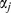. The classical BoVW model employs the “hard assignment” of a low-level descriptor to the closest codeword, and can be modeled by:
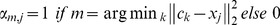
(1)where 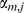
is the 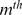 component of the encoded descriptor.

Recent publications [15], [34], however, suggest that “soft” assignment schemes, which allow degrees of association between the low-level descriptors and the elements of the codebook, work better, avoiding both the boundary effects and the imprecision of hard assignment [34].

The pooling step takes place after coding, and can be represented by a function 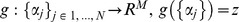. The classical BoVW corresponds to a “counting of words” (called sum-pooling) and can be modeled as:
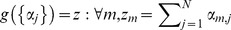
(2)


This simplistic pooling has been criticized, and taking the maximum activation of each codeword (in a scheme aptly named max-pooling) is often much more effective [35]:
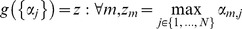
(3)


The vector 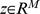 obtained from pooling is the BoVW representation, which is used for classification.

There are a number of choices to normalize the BoVW vector. For example, in the classical BoVW scheme, 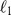-normalization is often employed to turn a vector of occurrences into a vector of relative frequencies.

The methods section outlines how the traditional BoVW models can be modified to improve discrimination as part of developing DR-related lesion detectors. In addition, it discusses how to combine the classification outcomes of different detectors in order to obtain a more global decision for an image.

## Materials and Methods

This section provides a complete description of the proposed technique. First a detailed overview of each conceptual aspect of technique is discussed; then, a precise procedural description of the scheme, detailing all steps and parameters is provided. The scheme proposed here employs a two-tiered image representation based upon the extraction of low-level local features from the images, and then the aggregation of those local features into mid-level BoVW features. Finally, the BoVW features are used as input to a maximum-margin SVM classifier [36].

### BoVW-based Representation

The mid-level BoVW representation is the main contribution of this paper and outlined here. Several BoVW-based representations have been proposed in the literature [16], [18], [38]. However, the methods discussed in these papers do not explore and compare the different possible implementations associated with BoVW-based representations nor do they present any elaborate discussion on the rationale for using the representations proposed therein.

BoVW-based representation rests upon several possible choices that have to be made for low-level feature extraction, type of codebook, coding and pooling when applying this method to image classification. The factors considered for this research are listed below and explained in the remainder of this section:

- **Low-level feature extraction**: mid-level BoVW features depend upon low-level features. The features used for low-level feature extraction have a large impact on subsequent performance of the classifier. Two low-level BoVW feature extraction possibilities (factor levels) are **sparse features**, based upon the detection of salient regions or points-of-interest; and **dense features**, sampled over dense grids of different scales;
- **Choice of codebook**: “codebook learning” was performed by a k-means clustering over features chosen **at random** from a training set of images. An alternative **class-aware** factor level is also proposed;
- **Coding**: For this factor, three levels were compared:
  - **Hard assignment**: associates each descriptor fully and only to its closest codeword in the codebook, (Eq. 1). The advantage of these schemes is the sparsity of the codes; the disadvantages are that they are subject to imprecision and noise when the descriptors fall in regions close to the limit between the codewords in the feature space. This scheme was explored in previous work for detecting DR-related lesions [16]–[18].
  - **Soft assignment**: there are several “soft” assignment schemes to deal with the deficiencies associated with hard assignment. The option employed here was *codeword uncertainty* [34], which has not been explored as a DR-related lesion detector but is generally considered the most effective for other classification tasks:
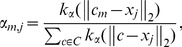
(4)where 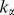 is the Gaussian kernel.
  - **Semi-soft assignment**: soft assignment solves the boundary effects of hard assignment, but creates too dense codes. A “semi-soft” scheme is often more desirable. One such scheme, designed specially for the DR-related lesion detection, is described below.
- **Pooling:** For the pooling step, both the traditional **sum**-pooling (Eq. 2) and the more recent **max**-pooling, described in Eq. 3, are employed. The pooling step is considered one of the most critical for the performance of BoVW representations, and max-pooling is considered an effective choice [15], [33], [35].

In all cases a 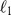-normalization on the final BoVW vector was used.

### Semi-soft Coding

The semi**-**soft coding tries to combine the advantages of both hard and soft assignments, i.e., avoiding the boundary effects of the former, and the dense codes of the latter. The main idea is to perform a soft assignment, but just to the codewords that are the closest to the descriptor, keeping all others at zero. This concept can be translated into many designs of which two were used for this research:

- only the closest codeword is activated;
- the activation is proportional to the inverse of the distance between the codeword and the descriptor.

Therefore, the generated codes are very sparse. On the other hand, the effect of the descriptors is “felt” even at relatively long distances (compared to exponential decay of a Gaussian kernel as in (4)). The scheme has the advantage of requiring no parameters.

The coding function can be described as:
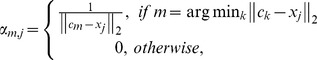
(5)


### Class-aware Codebook

Rocha et al. [16] proposed employing a “double codebook”, extending the usual scheme in a class-aware fashion, especially adapted for DR-related lesions. This is possible because, in addition to the training images being annotated for each lesion, the regions where the lesions appear are also identified (usually 2 to 5 per image from affected patients).

**Figure 2:**
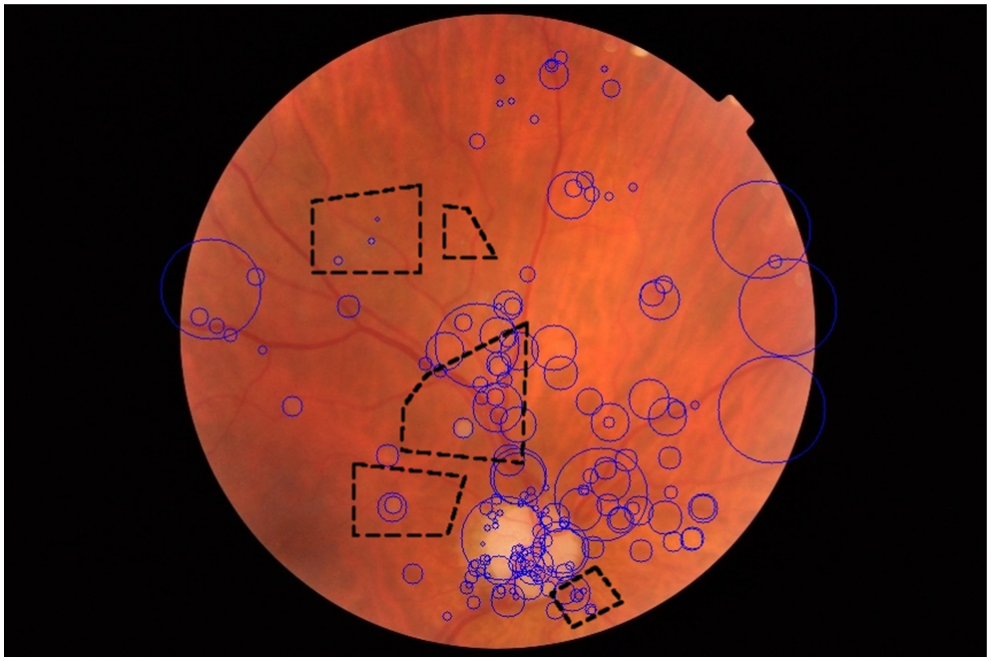
Regions of interest (dashed black regions) and the points of interest (blue circles). Points of interest falling within the regions marked by the specialist are considered for creating the class-aware codebook – half of the codebook is learned from local features sampled inside the regions marked as lesions, and half the codebook is learned from local features outside those regions.

Using the class-aware codebook ensures a sufficient number of codewords representing the appearance of the lesion structures. Because the lesion areas are relatively small, a non-class-aware codebook tends to be dominated by codewords representing healthy regions. During the coding phase, a good codebook is important, as the local feature vectors need to be assigned to the components of the mid-level feature vector in a way that allows discriminating the positive and negative classes. Having very few codewords for the lesion structures reduces this discriminating power. Selection of feature vectors is usually employed for general-purpose visual recognition – but in those tasks, recognition does not hinge on such subtle differences, as is the case for DR-related lesions. The scheme can be employed for both dense and sparse low-level descriptors, and is illustrated for the latter case in Figure 2.

The class-aware scheme works by creating two independent codebooks, one from descriptors sampled from regions marked as containing lesions by the specialist, and one from descriptors outside those regions (which includes images from healthy patients). Then, two independent 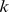-means clustering methods are performed, each with 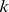 corresponding to half the size of the desired codebook. After the clustering is finished, the two sets of centroids are simply concatenated, generating a codebook of the desired size.

### The Procedure in Detail

*Creation of the training model for one type of lesion:*

1. Factor: **Low-level feature** ***detection*** –
  - Factor level: **dense** – on each image, patches are selected on a dense grid using radii of 12, 19, 31, 50, 80, 128 pixels. These radii are used both as scale and as the vertical/horizontal sampling steps of the grid;
  - Factor level: **sparse** – on each image, the SURF algorithm version 1.0.9, released by Bay et al. [39], is used to detect salient patches. Compared to the alternative SIFT [40], SURF is faster and has shown superior results in previous evaluation studies for DR-related lesion detection [16], [18], [38]. SURF sensitivity parameters are pre-tuned to detect 400 points of interest (PoIs) per image (after the filtering of step 2) on average, and to operate on twice the image resolution.
2. The edge of the retina in the images is found using a threshold and features falling outside the threshold are discarded.
3. *Low-level feature description*: SURF is used to create a feature vector for each detected point of interest. The algorithm is parameterized to operate on twice the image resolution and to extract 128-dimensional extended feature vectors instead of the default 64-dimensional ones.
4. Using the annotations of lesion regions provided by the medical specialists, two sets of feature vectors are found: “lesion” and “normal” (in our experiments, and average of 2,820 vectors for the lesion features and 19,710 vectors for identification of normal features were found).
5. Independently, a 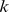-means clustering is employed on each set “lesion” and “normal”, using Euclidean distance, for 200 rounds or until convergence. For all treatments using **sparse** low-level feature detection 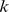 = 250, and for all treatments using **dense** feature detection 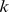 = 750. The larger codebook for dense extraction was an attempt to improve the results of dense extraction by considering a finer codebook that would be able to accommodate the extra amount of features being extracted;
6. The two sets of centroids are concatenated to form a codebook of 2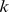 vectors;
7. Factor: **BoVW-based representation** –
  - Factor level: **hard assignment** – for each image, the BoVW is created by encoding its low-level feature vectors according to Eq. 1, and pooling using sum-pooling;
  - Factor level: **soft assignment** – for each image, the BoVW is created by encoding its low-level feature vectors according to Eq. 4, and pooling using max pooling. The standard-deviation employed in the Gaussian kernel was 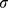 = 45, a value derived from observing a population of distances between pairs of SURF descriptors in a very large dataset of images independent from the ones used in this work;
  - Factor level: **semi-soft assignment** – for each image, the BoVW is created by encoding its low-level feature vectors according to Eq. 5, and pooling using max pooling;
8. The BoVW feature vectors, together with the medical specialists annotations into positive (with lesion) × negative (normal) classes are used to train an SVM model with a Gaussian kernel using LibSVM [37]. The classifier parameters C (the margin “hardness”, an inverse regularization parameter) and 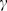 (the standard deviation of the kernel) are found by cross-validation, using the default built-in grid-search fine-tuning algorithm.
9. The output training model is composed of the midlevel codebook created at steps 4–6 and the classifier model created at step 8. No other data (images, low-level features, etc.) has to be preserved in order to make the classification step possible. One training model is created for each lesion, varying the annotations used in steps 4 and 8.

The scales 12, 19, 31, 50, 80, 128 chosen in step 1(a), allow the characterization of both small lesions, such as superficial hemorrhages, and very large ones, such as cotton-wool spots. The smallest and largest scales were selected after surveying the lesions (manually) in training images and determining the smallest and largest size of the structures of interest. The intermediate steps were chosen to form (roughly) a geometric progression. This methodology is usually applied in Computer Vision [56], [57], although recent works on very large training datasets – containing up to millions of images – tend to favor fewer scales. For smaller datasets such as medical images, where accuracy is at premium, a more exhaustive analysis, with a greater number of scales, is feasible.

*Obtaining the classification scores for one type of lesion:*

1. The image to be classified is described following steps 1–3 and 7, using the learned codebook. It is important to employ the same treatment used in the creation of the model (e.g., if the model was created using dense low-level features and hard assignment, this same treatment must be used for the images to be classified);
2. Using the learned SVM model and the BoVW feature vector created in step 10, a classification score is obtained, allowing deciding whether the lesion is present.

## Experiments

### Data, Protocol and Metrics

The experiments were performed using three different retinal image datasets annotated by medical specialists:

- **DR1 dataset**, provided by the Department of Ophthalmology, Federal University of São Paulo (Unifesp). Each image was manually annotated by three medical specialists and all the images in which the three annotations agree were kept in the final dataset. The images were captured using a TRC-50X (Topcon Inc., Tokyo, Japan) mydriatic camera with maximum resolution of one megapixel (640×480 pixels) and a field of view (FOV) of 45°.
- **DR2 dataset**, provided by the Department of Ophthalmology, Federal University of São Paulo (Unifesp) but images annotated by two medical specialists (none of them worked on the DR1 dataset). The dataset was captured using a TRC-NW8 retinograph with a Nikon D90 camera, creating 12.2 megapixel images, which were then reduced to 867×575 pixels for accelerating computation.
- **Messidor dataset**, captured in three different French ophthalmologic departments. There are three subsets, one for each department. The images were captured using a Topcon TRC-NW6 non-mydriatic retinograph with a 45° field of view, at the resolutions of 1,440×960, 2,240×1,488 or 2,304×1,536 pixels.

Both DR1 and DR2 datasets are publicly available under accession number 10.6084 and URL http://dx.doi.org/10.6084/m9.figshare.953671. The datasets were collected in different environments with different cameras, at least one year apart and in different hospitals. The Messidor dataset is also available for the scientific community: http://messidor.crihan.fr. Image characteristics of the three datasets are given in Table 3.

**Table 3:** Annotation occurrences for the three datasets.

Lesion	DR1	DR2	Messidor
Hard Exudates (HE)	234	79	654
Superficial Hemorrhages (SH)	102	–	–
Deep Hemorrhages (DH)	146	–	–
Red Lesions (RL)*	–	98	226
Cotton-wool Spots (CS)	73	17	–
Drusen (D)	139	50	–
Other lesions, excluding above	–	71	–
All lesions**	482	149	654
Normal (no lesions)	595	300	546
All images	1,077	520	1,200

*“Red Lesion” is a more general annotation that encompasses both SH and DH, besides microaneurysms.

**The lesions do not sum to this value because an image can present several types of lesion at once.

All experiments were performed using a cross-dataset protocol, an important precaution in the design, since in clinical practice the images that need to be classified have rarely the same image specification (camera, resolution, operator, FOV) than the images used for training. The datasets were collected in very different environments with different cameras, at least one year apart and in different hospitals. The entire DR1 dataset was employed as the training dataset. The DR2 and Messidor datasets were then employed for testing. The cross-dataset protocol poses experimental design challenges, because of the different standards used in the annotations of the three datasets. In DR1, images are annotated with the specific tags *deep* and *superficial hemorrhage*. In DR2, only the general *red lesion* tag is employed. In Messidor, the images are annotated not only for the presence of the lesions, but also for the severity, evaluating the number of microaneurysms and hemorrhages (red lesions), the presence or absence of neovascularization (not evaluated in this work), and the proximity of the exudates to the macula. In order to make the cross-dataset classification possible, and the joint statistical analysis of the two sets of experiments (DR2 and Messidor) feasible, we proposed correspondences in the annotations, detailed in Table 4.

**Table 4:** Composition of the cross-dataset training and testing.

	Train	Test	
Lesion	DR1	DR2	Messidor
Hard Exudates (HE)	234	79	654
Superficial Hemorrhages (SH)	102	–	–
Deep Hemorrhages (DH)	146	–	–
Red Lesions (RL)*	180	98	226
Cotton-wool Spots (CS)	73	17	–
Drusen (D)	139	50	–

*The annotations SH and DH are added to form the training set in DR1, summing 180 images due to the overlap.

The DR2 dataset has an additional annotation indicating the need for referral by the patient for follow-up by an ophthalmologist in the following 12 months after retinal assessment. Details about the annotation are presented in *Fusion (referral vs. non-referral classification)*. The dataset is freely available through FigShare repository, under accession number 10.6084 and URL http://dx.doi.org/10.6084/m9.figshare.953671.

To quantify precisely the performance of the proposed method and enable reliable comparisons, we employed receiver operating characteristic curves (ROCs), which plot the compromise between specificity (few false positives) and sensitivity (few false negatives). To quantify performance as a single scalar, the area under the ROC curve (AUC) was applied. Since the classifier can trade specificity for sensitivity, the AUC gives a better overall performance measure than any particular point of the specificity-sensitivity metrics.

The source code implementing the technique, as well as the scripts performing the experiments are available through GitHub: https://github.com/piresramon/retina.BoVW.plosone.git. The source code and scripts are written in Python, and use python.numpy 1.3.0, python.matplotlib 0.99.1.1, and python.scipy 0.9.0 as dependencies. The adopted license for the code is GPLv3. Slight differences in the feature extraction phase are expected since some parts of the code rely on other publicly available libraries and they are in constant update.

## Results

The detailed results are presented in Tables 5 and 6, which show the AUCs obtained for each lesion with the DR2 and Messidor datasets.

**Table 5:** Accuracy for Training with DR1, Testing with DR2.*

	Sparse features			Dense features		
	Hard	Semi-soft	Soft	Hard	Semi-soft	Soft
Hard Exudates (HE)	93.1	**97.8**	95.5	94.5	95.6	95.6
Red Lesions (RL)	92.3	**93.5**	87.1	89.1	90.6	89.9
Cotton-Wool Spots (CS)	82.1	**90.8**	84.9	84.5	90.4	90.3
Drusen (D)	66.5	82.8	62.6	**84.1**	82.5	75.5

*AUC in %; best accuracy is shown in bold.

**Table 6:** Accuracy for Training with DR1, Testing with Messidor.*

	Sparse features			Dense features		
	Hard	Semi-soft	Soft	Hard	Semi-soft	Soft
Hard Exudates (HE)	64.4	70.3	66.2	**70.5**	70.0	70.0
Red Lesions (RL)	77.4	83.1	76.6	**85.2**	85.1	82.5

*AUC in %; best accuracy is shown in bold.

Results shown in Tables 5 and 6 suggest that the best configuration of the BoVW for each lesion (and dataset) are the proposed semi-soft coding on sparse features, except for the drusen, where semi-soft coding performs best with dense features. We believe that the good performance of dense features on Messidor is due to the presence of very challenging images (patients with very early DR signs, showing very few lesions). However, the results show show that the semi-soft coding scheme works well on the Messidor dataset when associated either with sparse features or dense features.

Such local case-by-case analysis, however, fails to account for random effects. A less naïve analysis must take into account all results across BoVW parameters, datasets and lesions. The goal in DR classification is to obtain the overall best configuration for the BoVW, if such configuration can be found with confidence. The DR2 and Messidor datasets provide different annotation standards, with the former having annotations for all four levels of lesions, but the latter having annotations only for hard exudates (HE) and red lesions (RL). This presents a challenge for performing (and to interpreting) such unbalanced experimental designs and separate balanced studies were performed: one considering only DR2 and all four lesions; and another for both test sets, but with only HE and RL lesions.

**Figure 3:**
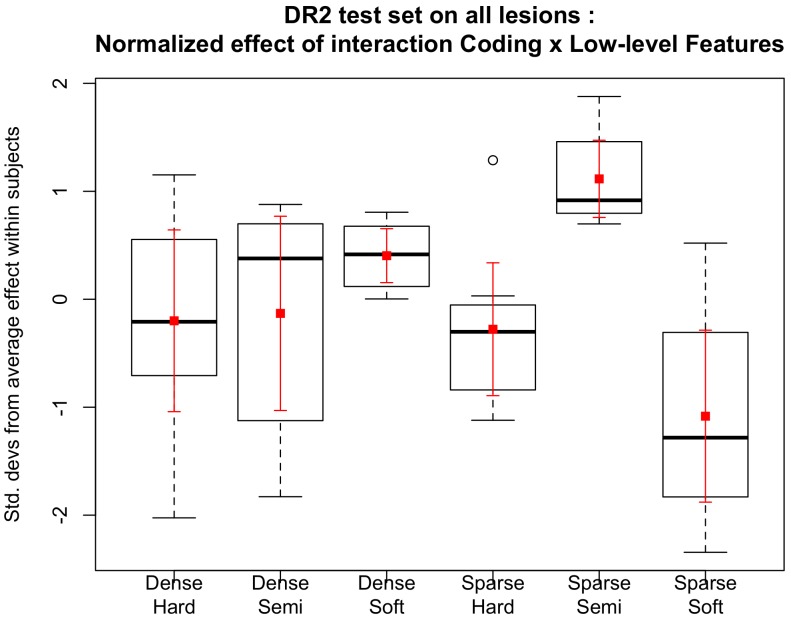
Standardized AUCs per lesion, for six combinations of feature extraction and coding (horizontal axis). In the box-plots (black), the whiskers show the range up to 1.5× the interquartile range, and outliers are shown as small circles. Averages (small squares) and 95%-confidence intervals (error bars) are also shown, in red, for the same data. The strong synergy between sparse feature extraction and semi-soft coding is evident: it has consistently improved results for all lesions, while the other combinations improve the results of some lesions at the cost of decreasing it for other lesions (as shown by the spread of the standardized effects in the vertical axis). This plot is based on a balanced design with the DR2 dataset and all lesions, the other balanced design with both datasets and two lesions show similar results.

The box-plot in Figure 3 illustrates, for each treatment, how much it improves or decreases the performance of the detection of the lesions, in comparison to the other treatments. As the lesions and datasets vary widely in difficulty, and we are interested in determining a treatment (combination of factor levels) that performs globally better than the others, we analyzed the normalized impact on the AUC of each factor. In order to do that, for each combination of lesion–dataset, we normalized the AUCs (subtracting the mean and dividing by the standard deviation of AUCs for that combination). More formally, the procedure takes each specific lesion ℓ, computes the mean AUC µ_ℓ_ for all treatments on that lesion, computes the standard deviation of those AUCs σ_ℓ_, and then, if the AUC of a specific treatment on that lesion is *β*_ℓ_, the normalized AUC will be 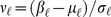. Therefore, Figure 3 shows, graphically, those standardized effects. The correct interpretation of the box-plot shows, for example, that the treatment “sparse–semi-soft” is, on average for all lesions on DR2, one standard deviation above the mean of AUCs obtained by all treatments, i.e., 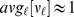.

The synergy between sparse feature extraction and semi-soft coding for DR-lesion classification can be appreciated in the box-plot of Figure 3. Remark that most combinations of feature extraction and coding function have a wide distribution of standardized effect, meaning that they improve the detection of some lesions at the cost of decreasing the performance of others. In contrast, sparse feature extraction and semi-soft coding offer consistently improved results.

In order to obtain quantitative results, we have also performed a factorial ANOVA that formalizes the same experimental design used on Figure 3. The following factors (and levels) were employed:

1. low-level feature extractor (Sparse, Dense),
2. coding (Soft, Semisoft, Hard), and
3. test dataset (DR2, Messidor)

with repeated measures for each lesion (HE, RL, CS, D) and all errors measured within-subjects (the subjects are each individual combinations of lesion and dataset). To remove the strong scaling effect of the lesions and datasets, each subject was independently standardized by subtracting the average and dividing by the standard deviation, as explained above.

The analysis on the DR2 subset indicated an important interaction effect between the choice of Low-level Features and Coding (*p* = 0.007). The main effect of Coding alone just fails significance (*p* = 0.062), and all other effects and interactions are non-significant. These factors have a significant interaction effect due to the two low-level feature extractors providing better results with different coding schemes (Table 5). The analysis on the other data subset, with both test datasets and only HE and RL lesions, shows similar results, with significantly better outcomes for the sparse+semi-soft combination (*p* = 0.011).

A crucial factor of the current study is the validation protocol. The training and testing was performed using distinct datasets, exploring the cross-validation protocol, which is more robust than the 5-folds cross-validation used previously. Despite using this stricter protocol of different datasets for training and testing, our results for hard-sum compare well to previous results obtained by our team.

### Fusion (Referral vs. Non-referral Classification)

One important challenge in DR-related lesion detection is making a decision about the necessity of referral to a specialist, instead of only indicating the presence of particular anomalies [41]–[44]. This section is an extension of a previous work, in which we proposed a method that recommends referring a patient with diabetes for diabetic retinopathy assessment based on the image classification outcome [44].

As in our previous publication [44] this work also investigates the need for referral. However, now we prioritized the development of a new algorithm aiming at improving the accuracy of the individual lesion detectors and consequently also the assessment of the referral classifier. In addition to all the contributions mentioned in the Introduction, we highlight the original innovation regarding the proposed semi-soft pooling technique, which is highly effective for common DR-related lesions such as red and white lesions as well as for the hard-to-detect lesions, such as cotton-wool spots and drusen, creating a better representation that can be explored for referral assessment.

The approach taken here was to use the best individual detectors i.e., the ones that employ sparse feature extraction with semi-soft assignment, and employing fusion techniques in order to make a final decision. For comparison the coding and pooling approaches employed in [44], hard-sum and soft-max assignments, both with the sparse features in the low-level extraction were implemented.

The most advanced way to perform the fusion is using a meta-classification approach. This referral-decider operates on high-level features obtained from a vector of scores consisting of the individual lesion detectors. The referral-decider is then trained using independent annotations.

Considering the sparse feature detection as the best choice to detect DR-related lesions, for each coding/pooling technique explored and proposed in this work, we employed all six detectors trained on the DR1 dataset. The scores of the detectors are then used to compose the feature-vectors of a second-layer classifier.

Note that one important parameter for the individual classifier is their operational setup (the sensitivity-specificity compromise). This parameter can vary widely. In order to make the meta-classification feature-space more stable, a 5×2-fold cross-validation design was applied [45] for validating the results using meta-classification of the DR2 dataset: the set is divided in half; one half is used for training and the other for testing; the procedure is repeated five times. Training and testing are carried out on the DR2 “same lesions” scenario, as described above. The training part is used for finding the best operational points for each detector learned from the DR1 dataset as well as for finding the classification parameters regarding the meta-classification SVM model (second layer classifier).

DR2 contains 98 images annotated as referable and 337 images labeled as non-referable. For annotation, the specialists usually consider the number and the location of DR-related lesions, among other factors. The Messidor dataset could not be used in assessment for referral as it lacks the needed annotations.

**Figure 4:**
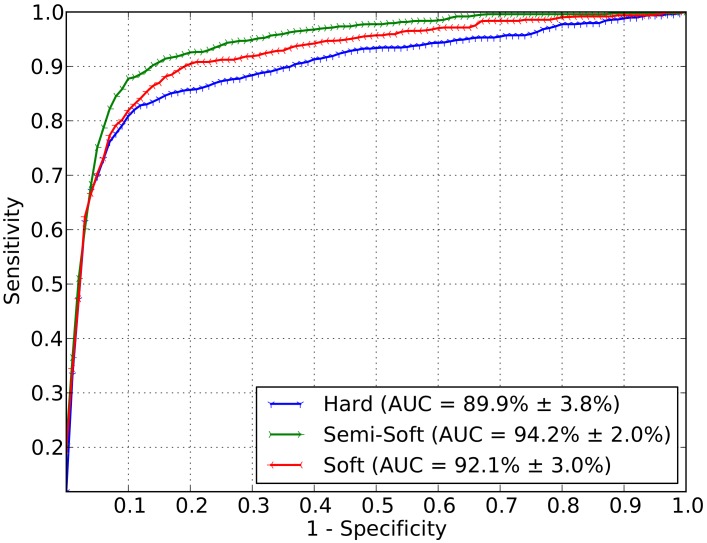
Final decision for necessity of referral. The decision is based upon meta-classification using the scores of the individual lesion detectors as features. The meta-classifier is trained and tested on the DR2 dataset.

Figure 4 shows the ROC curves that express the mean and the standard deviation obtained using meta-classification. The area under the curve is equal to 89.9
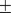
3.8% for hard-sum as the assignment technique. Using the soft assignment technique associated to the max pooling, an AUC of 92.1
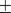
3.0% was obtained. The semi-soft approach, proposed in this work for DR-related lesion detection, outperformed the already known techniques (e.g., [16]) also for a higher-level classification stage with an AUC of 94.2
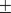
2.0%.

To evaluate the significance of the referral results, the Friedman test was employed followed by the Nemenyi post hoc analysis, which indicated that the semi-soft coding analysis was superior to the others for referring accuracy (p = 0.007) [46], [47].

## Discussion

Automated lesion detection has a huge potential to facilitate the identification of diabetic retinopathy progression, and the access to care, for rural and remote communities, providing a screening tool able to determine which patients need to be referred to specialists. Moreover, by providing an accurate detection of lesions at the early stages of DR, automated lesion detection has the potential to reduce treatment costs and improve prognosis.

Previous research on the detection of diabetic retinopathy related lesions obtained satisfactory results for the detection of single DR-related lesions. However, the detection of different lesions normally relied upon the use of distinct image classification approaches based on specific properties of each lesion type. That made the detection of multi-lesion detection as part of DR progression a complex procedure, since it requires the implementation, parameterization and validation of multiple very distinct detection methods. On the other hand, recent advances in DR-related lesion detection based on bags-of-visual-words approach proposed an elegant unified approach for all DR-related lesions, with the additional advantage of bypassing the need of pre- and post-processing operations.

The state of the art in BoVW methods for DR-lesion detection was advanced by extending possible combinations of applying BoVW for detecting DR-related lesions in retinal images. We explored several combinations of alternatives for the extraction of low-level features, and the creation of mid-level representations pointing out important choices when designing a unified framework for detecting DR lesions.

One of the contributions in this paper is the proposal of a new semi-soft coding scheme, which explores the advantages of the most traditional hard-sum coding (sparse coding) as used in prior work for DR lesion detection [16] and soft assignments (which better deal with imprecisions and noise). As we show in the experiments, with ANOVA, the semi-soft coding associated with sparse feature extraction provides a good balance for designing an efficient and effective DR-related lesion detector. A comparison with Rocha et al.’s paper [16], in which the class-aware scheme is proposed for the detection of bright and red lesions exploiting the classical hard-sum approach, indicates that the current innovation significantly improves the outcome of DR lesion classification. Rocha et al. obtained AUCs of 95.3% and 93.3% for bright and red lesions respectively, whilst the current results obtained an AUC of 97.8% for bright lesions and 93.5% for red lesions from images of the DR2 data set. New findings from the current study include excellent results for two hard-to-detect DR lesions: cotton-wool spots (AUC = 90.8%) and drusen (AUC = 82.8%).

At least for the particular problem of DR-related lesion detection, the sparse feature extraction + semi-soft coding combination implemented for this work leads to a different conclusion to the current art on Computer Vision for general object recognition, in which dense sampling + soft assignment is consistently reported to give better results.

In this research, we prioritized the decision on need of consultation directly from the lesion detectors, instead of relying on intermediate steps of localizing individually the lesions, or assessing lesion severity. Given the best representation, we devised fusion techniques for defining whether the patient need or not be referred to a specialist. In this sense, using an elaborated fusion technique based on meta-classification (which seeks a pattern based upon the classification score confidences returned by each individual lesion detector), we achieved an AUC of 94.2%
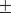
2.0% that outperforms other approaches and represents a step forward for automatic assessment of referral necessity. That result outperformed also the best one obtained in our previous work for referral vs. non-referral classification (93.4
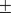
2.1%) [44].

Our work here did not aim at evaluating diabetic retinopathy severity, but at detecting the presence or absence of DR-related lesions, an active area of research (see [4], [44], [52], and [53], for some examples). The current protocols employed are not comparable with diabetic retinopathy classification protocols used in studies such as Early Treatment Diabetic Retinopathy Study (ETDRS) [49], and the Wisconsin Epidemiologic Study of Diabetic Retinopathy (WESDR) [48], because the aims of the current research were to identify images that required referral based on the presence of red and white lesions. It must also be noted that grading systems like ETDRS are somewhat complicated and pose a challenge for correct use in clinical situations [51]. That has motivated further recommendations, including the Australian Guidelines [50], which simplified the diabetic retinopathy classification.

The current contribution is important in a practical context, as it simplifies screening procedures, which can be carried out in rural and remote communities with the use of nonmydriatic cameras, and without the presence of a specialist. The sensitivity and specificity obtained in our experiments are high and fall within international guidelines for DR detection accuracy for multi-lesion detection [50], [54], [55]. Necessity of referral however does not require determining the exact location of either red or white lesions. The current protocol however does provide the type of lesion present in the image. Presence of either or both these lesions would require further investigation by an ophthalmologist.

Although we have not directly focused on the computing time (or processing cost) herein, we note that none of the techniques tested as part of the current research is much expensive. To put the figures into context, the training step (which is performed once and offline) takes a few hours (typically less than six hours), and the testing step (performed online for each patient image) takes at most two minutes. Those times are for a computer with a 2.6 GHz processor, four computing cores, and 16 GB of RAM. To offer a qualitative idea, the combinations that employ dense extraction for the low-level descriptors are slower than the ones that employ sparse extraction; and, for the coding, the hard assignment is the fastest, the soft assignment is the slowest, and the semi-soft falls in-between.

Finally, the discovery of the best method for effective DR-related lesion detection opens the opportunity for deploying the sparse technique with semi-soft coding to other applications. A possible future work consists of identifying the precise location of the lesion, as well as the size and quantity of different lesion types associated with DR, and defining the degree of DR severity of a patient as early, mild, moderate nonproliferative retinopathy and proliferative.
